# Biofabrication and Characterization of Fluorapatite-Coated Poly(lactic-co-glycolic acid) Microscaffolds: Physicochemical Properties and Human Dental Pulp Stem Cell Responses

**DOI:** 10.3390/jfb17090479

**Published:** 2026-09-19

**Authors:** Saya Hadi Raouf, Varvara Platania, Argyro Lamprou, Youri Arntz, Iryna Lysova, Diyar Khalid Bakr, Niaz Hamaghareeb Hamasaeed, Mutlu Özcan, Isaac Maximiliano Bugueno

**Affiliations:** 1 College of Dentistry, Hawler Medical University (HMU), Kurdistan Region, Erbil 44001, Iraq; saya.raouf@hmu.edu.krd (S.H.R.); diyar.bakr@hmu.edu.krd (D.K.B.); nyaz.hamagharib@hmu.edu.krd (N.H.H.); 2 Orofacial Development & Regeneration Unit, Center for Dental Medicine, Plattenstrasse 11, 8032 Zurich, Switzerland; varvara.platania@zzm.uzh.ch (V.P.); argyro.lamprou@zzm.uzh.ch (A.L.); 3 Inserm UMR_S 1121, Laboratoire de Biomatériaux et Bioingénierie, Université de Strasbourg, 67085 Strasbourg, France; youri.arntz@unistra.fr (Y.A.); iryna.lysova@unistra.fr (I.L.); 4 Clinic of Masticatory Disorders and Dental Biomaterials, Center for Dental Medicine, University of Zurich, 8032 Zurich, Switzerland; mutlu.ozcan@zzm.uzh.ch; 5 Institut de Génétique et de Biologie Moléculaire et Cellulaire (IGBMC), INSERM U1258, CNRS UMR7104, Université de Strasbourg, 67400 Illkirch-Graffenstaden, France

**Keywords:** fluorapatite, PLGA, dental pulp stem cells, tissue engineering, biomaterials, regenerative dentistry, microscaffolds

## Abstract

Biodegradable polymeric scaffolds incorporating bioactive mineral phases are a promising approach for dentin-pulp tissue engineering. Although poly(lactic-co-glycolic acid) (PLGA) microparticles have been widely used as scaffolds due to their biocompatibility and tunable degradation profile, their inherent bioactivity is limited. Furthermore, fluorapatite (FAP), a fluoride-substituted apatite ceramic, exhibits enhanced chemical stability and mineral-related properties that may be useful for regenerative biomaterial design. In this study, we investigated the effect of nano-FAP functionalization on the physicochemical properties of porous PLGA microscaffolds and their interaction with human dental pulp stem cells (hDPSCs). Porous PLGA microscaffolds were fabricated using a double-emulsion solvent evaporation method and subsequently functionalized with FAP suspensions ranging from 0.1 to 5 mg/mL (0.01–0.5% *w*/*v*). The scaffolds were evaluated using scanning electron microscopy (SEM), Fourier-transform infrared spectroscopy (FTIR), electrical conductivity measurements, cell viability assays, and immunofluorescence. Lower and intermediate FAP concentrations-maintained surface pore accessibility and supported hDPSC viability, whereas the highest concentration (5 mg/mL; 0.5% *w*/*v*) reduced visible surface pore size and showed less favorable cellular responses. The 2.5 mg/mL (0.25% *w*/*v*) FAP condition provided the most favorable overall balance among the evaluated physicochemical and biological parameters. These preliminary in vitro findings support further investigation of FAP-functionalized PLGA microscaffolds in advanced three-dimensional and in vivo models.

## 1. Introduction

The restoration of the dentin-pulp complex remains one of the most significant challenges in modern regenerative dentistry. Teeth are frequently affected by infectious diseases, such as dental caries, and traumatic injuries, which often lead to irreversible dentin and pulp vitality loss [1,2]. Traditional restorative approaches, such as root canal treatments and vital pulp therapy primarily use inert synthetic materials that fail to properly restore the biological structure, functionality, and sensitivity of the native dentin-pulp complex [3,4]. Consequently, the field of regenerative dentistry has shifted its focus toward tissue engineering approaches that aim to regenerate the dentin-pulp complex by combining stem cells, bioactive scaffolds and signaling molecules that promote endogenous repair mechanism [5].

Human dental pulp stem cells (hDPSCs) have been widely used across regenerative dentistry due to their high versatility, significant proliferative capacity, multi-lineage differentiation potential, and ease of accessibility from third molar extractions [4,6,7,8,9]. Importantly, their ability to differentiate toward odontoblast-like and mineralizing phenotypes make them a relevant cellular model for evaluating biomaterials intended for dentine-pulp therapies. Equally important is the selection of an appropriate scaffold to support cells growth, proliferation, adhesion and differentiation [10]. Notably, poly (lactic-co-glycolic acid) (PLGA) is a clinically approved biodegradable synthetic polymer with excellent biocompatibility and tunable mechanical properties, that make it an ideal candidate for the biofabrication of scaffolds in dental applications [11,12,13,14]. Accordingly, hDPSCs were selected in the present study as a biologically relevant in vitro model to evaluate their interactions with functionalized PLGA microscaffolds and their potential to support dentin-pulp regenerative applications.

Although PLGA possesses favorable mechanical properties as a scaffold and delivery system, its ability to induce intrinsic biological responses is limited [15]. Therefore, the functionalization of PLGA scaffolds with bioactive ceramic nanoparticles has been proposed to enhance their biological performance in dental applications [16].

Hydroxyapatite (HA) has been widely used as a bioactive ceramic in tissue engineering due to its chemical similarity to the mineral component of bone and dentin. However, fluorapatite (FAP) has attracted increasing attention as an alternative biomaterial due to its improved physicochemical properties [17,18]. Fluorapatite (FAP) is formed by the substitution of hydroxyl groups in hydroxyapatite with fluoride ions, which results in enhanced crystallinity, increased structural stability, lower solubility, higher chemical stability, and greater resistance to acidic degradation [18,19]. Moreover, fluoride ions have been reported to stimulate mineralization and promote cell adhesion, proliferation, and odontogenic differentiation [20]. These properties suggest that incorporation of fluorapatite nanoparticles into PLGA scaffolds can enhance bioactivity and provide biochemical cues that support hard tissue regeneration. Nevertheless, the application of fluorapatite-functionalized PLGA microscaffolds in the context of hDPSC-mediated regeneration remains elusive. In particular, there is a lack of information regarding the effect of fluorapatite surface modification on scaffold physicochemical properties and subsequent cellular responses.

The aim of this study was the biofabrication and characterization of FAP-coated PLGA microscaffolds and the evaluation of their in vitro biological performance in hDPSC cultures. Unlike previous studies that have primarily investigated apatite-containing bulk scaffolds, hydrogels, or other composite systems, the present work focuses on porous PLGA microscaffolds functionalized after fabrication using a simple immersion-based FAP surface-coating strategy. A range of FAP concentrations was systematically evaluated in terms of surface morphology, FTIR characteristics, electrical conductivity, cytocompatibility, metabolic activity, and early cell-marker expression. FAP functionalization enhanced scaffold bioactivity while maintaining a porous microarchitecture supportive of hDPSC viability and cell–material interactions. Importantly, the observed biological responses should be interpreted as resulting from the combined physicochemical effects of the FAP-modified surface, including mineral-phase presentation, changes in surface architecture, and potential ionic contributions; since fluoride-ion release was not directly quantified, these effects cannot be specifically attributed to released fluoride ions. Therefore, this concentration-resolved approach aimed to identify a formulation that provides an appropriate balance between mineral-related physicochemical properties, preservation of scaffold architecture, and hDPSC compatibility, rather than assuming that increasing FAP loading necessarily improves biological performance. This rationale is consistent with recent studies of multifunctional porous scaffolds demonstrating that regenerative performance depends on the combined effects of scaffold architecture, physicochemical functionality, and cytocompatibility, emphasizing the importance of formulation-specific optimization [21].

## 2. Materials and Methods

### 2.1. Fabrication of Porous PLGA Microscaffolds

Porous PLGA microscaffolds were fabricated using a double emulsion (W1/O/W2) solvent evaporation technique with gelatin as a water-soluble porogen, adapted from [13]. Briefly, PLGA (0.2 g; 50:50, Mw: 30,000–60,000, ref P219, Mowiol^®^, Sigma Aldrich/Merk, Darmstadt, Germany) was dissolved in 10 mL of dichloromethane (DCM) with ref 270997 (Sigma Aldrich/Merk, Darmstadt, Germany) under magnetic stirring in a chemical fume hood to obtain a 2% (*w*/*v*) polymer solution. In parallel, a 0.3% (*w*/*v*) polyvinyl alcohol (PVA) with ref 81383 (Sigma Aldrich) solution was prepared by dissolving PVA powder in distilled water 50 °C for 4–5 h under continuous stirring, followed by stirring at room temperature until complete dissolution was obtained. A 10% (*w*/*v*) gelatin solution ref G9391 (Sigma Aldrich) was then prepared by dissolving the appropriate amount of gelatin in the warm PVA solution at 40–50 °C.

For the primary emulsion (W1/O), 1 mL of the gelatin/PVA solution was added dropwise into the PLGA/DCM solution while being homogenized by sonication (20% pulse, frequency 10 × 2 min), with the sample kept in an ice bath to limit solvent evaporation. This W1/O emulsion was then slowly poured in a thin stream into 240 mL of ice-cold 0.3% PVA under continuous stirring at 200–250 rpm to form the secondary W1/O/W2 double emulsion. The suspension was stirred gently overnight in a fume hood to allow gradual evaporation of DCM and solidification of the microscaffolds.

Following washing, the already formed microscaffolds were subjected to a single freeze-drying (lyophilization) cycle using a Christ Alpha 1–4 LSC plus freeze-dryer (Martin Christ Gefriertrocknungsanlagen GmbH, Osterode am Harz, Germany) [13]. Freezing and drying were performed within the same lyophilization process at −55 °C under vacuum (0.47–0.63 mbar) for 6–8 h to obtain dry, porous PLGA microscaffolds suitable for subsequent characterization and cell-culture experiments.

### 2.2. Fluorapatite Loading to PLGA Scaffold

**Figure 1:**
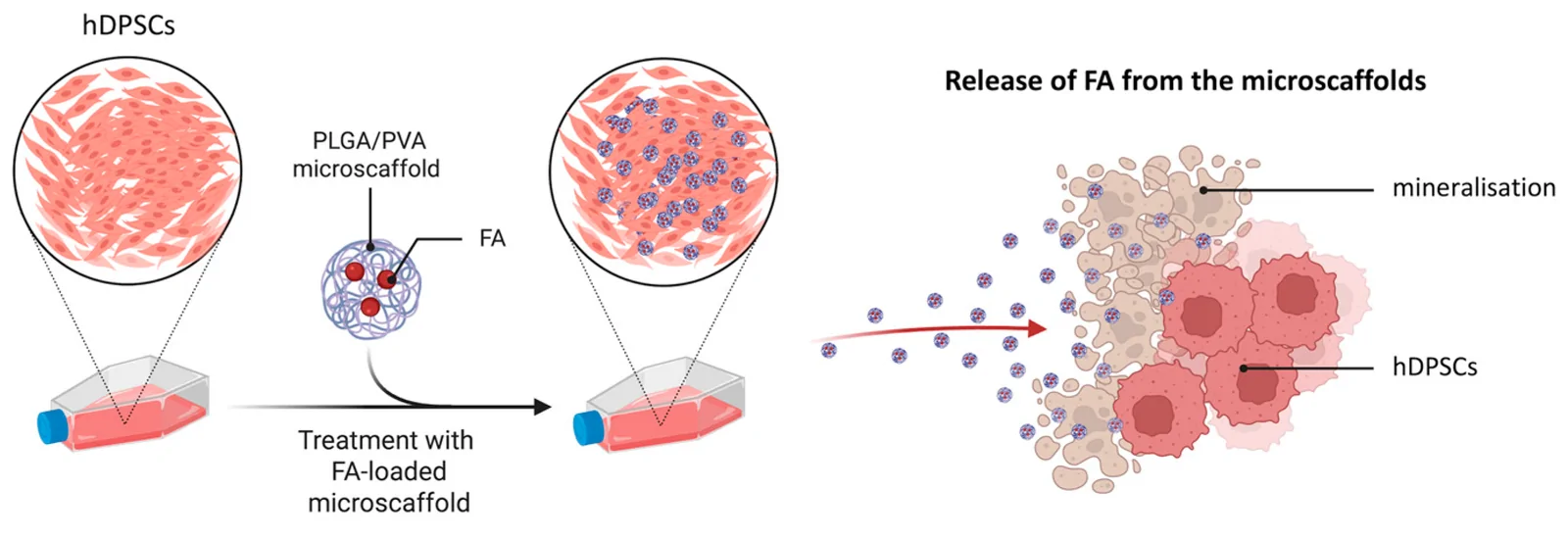
Schematic illustration of FAP surface-functionalization of porous PLGA microscaffolds for dental tissue engineering. Porous PLGA microscaffolds were fabricated and subsequently immersed in FAP suspensions of different concentrations to obtain FAP-functionalized microscaffolds for the evaluation of physicochemical properties and interactions with hDPSCs.

To enhance the bioactivity of the PLGA microscaffolds, a surface functionalization process was performed using FAP nanoparticles (Nanoshel LLC, Wilmington DE, United States). Based on previous studies [22,23], a range of FAP concentrations was initially explored; however, higher concentrations (10% *w*/*v* (100 mg/mL), 5% *w*/*v* (50 mg/mL), and 2.5% *w*/*v* (25 mg/mL)) showed poor dispersion and were excluded. The final concentrations used were 0.5% (*w*/*v*) (5 mg/mL), 0.25% (*w*/*v*) (2.5 mg/mL), 0.1% (*w*/*v*) (1 mg/mL), 0.05% (*w*/*v*) (0.5 mg/mL), 0.01% (*w*/*v*) (0.1 mg/mL), and 0% (*w*/*v*) (0 mg/mL), along with a control group consisting of pure PLGA microscaffolds without FAP (0% *w*/*v*) (Figure 1). For clarity, group labels mic_FAP_5, mic_FAP_2.5, mic_FAP_1, mic_FAP_0.5, and mic_FAP_0.1 refer to the FAP concentration of the loading suspension in mg/mL and do not represent the final FAP mass fraction incorporated into the microscaffolds. The actual amount of FAP adsorbed onto the microscaffolds was not quantified in the present study.

FAP suspensions were prepared in double-distilled water and maintained at 37 °C overnight to improve dispersion. Functionalization was achieved via an immersion-based adsorption method, where microscaffolds were incubated in FAP suspensions (~1:10 scaffold-to-solution ratio) to allow passive nanoparticle attachment. No sonication or chemical crosslinking was applied. The present study did not include a direct quantitative measurement of coating efficiency. SEM and FTIR were therefore used as complementary qualitative/semi-quantitative evidence of surface-associated FAP, while concentration-dependent heterogeneity and aggregation were considered when interpreting the morphology. Future studies should quantify coating efficiency and spatial uniformity using techniques such as elemental mapping and direct mineral mass/ion analysis.

### 2.3. Scanning Electron Microscopy (SEM) Characterization

Surface morphology, particle size, and visible surface pore size of PLGA and PLGA-FAP microscaffolds were evaluated by SEM using a Zeiss Gemini SEM 450 system (Oberkochen, Germany) at the University of Zurich. Dried samples (50–200 µm) were dispersed onto aluminum stubs covered with conductive carbon adhesive tabs and gently tapped to obtain a single-particle layer. Samples were sputter-coated with a thin conductive Pd layer (10 nm). SEM imaging was performed under high vacuum at low accelerating voltage (typically 1–3 kV; commonly 2 kV) using secondary electron detection (InLens and/or SE2) at a working distance of ~4–8 mm. Image acquisition was performed using the SmartSEM interface, and measurements were performed using the microscope facility tools and image-analysis software ImageJ/FIJI Version 1.54p (https://imagej.net/) and Imaris software (version 11.0, Oxford Instruments, Zurich, Switzerland). For quantitative surface pore-size analysis, at least three independent SEM fields and ≥50 clearly visible surface pores per group were analyzed under identical calibration settings. Pore diameter data are therefore interpreted as a two-dimensional measure of visible surface pore size and not as total scaffold porosity, pore volume, pore interconnectivity, or three-dimensional architecture. Data were expressed as mean ± standard deviation and analyzed using one-way ANOVA followed by Dunnett’s multiple comparison test, with mic_FAP_0 as the control (*p* < 0.05). These measurements represent two-dimensional diameters of clearly visible surface pores in SEM images and should not be interpreted as total three-dimensional scaffold porosity or interconnected pore volume.

### 2.4. Fourier Transform Infrared (FTIR) Analysis

FTIR spectroscopy was performed to characterize the chemical structure of PLGA and PLGA-FAP scaffold specimens and to identify spectral changes associated with FAP treatment. Spectra were recorded using an Anton Paar Lyza 7000 spectrometer (Anton Paar GmbH, Graz, Austria) equipped with a diamond ATR crystal over 4000–400 cm^−1^. Measurements were acquired in absorbance mode with 36 scans per sample at a spectral resolution of 4 cm^−1^. Functional groups were assigned according to established literature values. FTIR was used as supportive evidence for phosphate-containing apatite-related signals; it was not used to determine mineral crystallinity, elemental composition, spatial distribution, or to independently establish the mineral phase as fluorapatite.

### 2.5. Electrical Conductivity Test

Electrical conductivity was measured to evaluate the ionic contributions of fluorapatite suspensions and FAP-functionalized PLGA microscaffolds. Measurements were performed using a conductivity meter (Mettler Toledo^®^ Lösungen, Greifensee, Switzerland), equipped with a standard conductivity probe. The instrument was calibrated prior to each measurement set according to the manufacturer’s instructions using standard conductivity calibration solutions. Initial FAP stock suspensions used for microscaffold functionalization were prepared in double-distilled water (ddH_2_O) at the following concentrations: 0.5% (*w*/*v*) (5 mg/mL), 0.25% (*w*/*v*) (2.5 mg/mL), 0.1% (*w*/*v*) (1 mg/mL), 0.05% (*w*/*v*) (0.5 mg/mL), 0.01% (*w*/*v*) (0.1 mg/mL), and 0% (*w*/*v*) (0 mg/mL).

The electrical conductivity of each FAP suspension was measured directly in ddH_2_O immediately after preparation, ensuring homogeneous dispersion prior to analysis. ddH_2_O alone was used as an additional reference control. PLGA microscaffolds functionalized with FAP at the same concentration range (0.5%, 0.25%, 0.1%, 0.05%, 0.01%, and 0% *w*/*v*) were also evaluated. For these measurements, microscaffolds were resuspended in ddH_2_O at a standardized concentration of 1% (*w*/*v*) prior to conductivity analysis. Each suspension was gently vortexed to ensure uniform dispersion before measurement. The conductivity probe was immersed directly into the suspension, ensuring full electrode coverage. The probe was rinsed with ddH_2_O between measurements to prevent cross-contamination. Conductivity values were recorded in µS/cm after signal stabilization.

All measurements were performed in triplicate, and the results were expressed as mean ± standard deviation. Electrical conductivity data were analyzed using GraphPad Prism (version 8.0.2, GraphPad Software, San Diego CA, USA). For FAP suspensions in ddH_2_O, statistical comparisons among groups were performed using one-way analysis of variance (ANOVA) followed by Dunnett’s multiple comparison test, with ddH_2_O (0 mg/mL FAP) serving as the control group. For FAP-functionalized microscaffolds resuspended in ddH_2_O, a separate one-way ANOVA with Dunnett’s post hoc test was applied, using the 0% FAP microscaffold group (PLGA without FAP) as the control. A *p*-value < 0.05 was considered statistically significant.

### 2.6. Cellular Protocol and Seeding

#### 2.6.1. hDPSC Culture

Human DPSCs were isolated at the Centre of Dental Medicine, University of Zurich (Zurich, Switzerland). Written informed consent was obtained from all donors prior to the collection and use of their biological material for research purposes. All samples were anonymized before their use in the present study. All experimental procedures were conducted in accordance with protocols approved by the Ethics Committee of the Canton of Zurich (reference number: 2012-0588), with the latest authorization granted on 7 November 2023 (reference number: A230123-00).

hDPSCs were cultured and expanded following established protocols at the University of Zurich. Cryopreserved hDPSCs were rapidly thawed in a 37 °C water bath until only a small ice crystal remained, then immediately diluted in 10 mL of pre-warmed culture medium and centrifuged at 1200 rpm for 5 min at 25 °C to remove residual cryoprotectant. The resulting cell pellet was resuspended in 10 mL of expansion medium consisting of α-MEM (Gibco, Thermo Fisher Scientific, Reinach BL, Switzerland) supplemented with 10–15% fetal bovine serum (FBS), 1% penicillin-streptomycin, and 2.5 µg/mL fungizone to prevent fungal contamination. Cells were maintained in T75 flasks at 37 °C in a humidified atmosphere with 5% CO2. The medium was replenished every three days.

Upon reaching 85–100% confluency, cells were detached using 0.1% Trypsin-EDTA for 3 min at 37 °C. The enzymatic reaction was neutralized with a triple volume of culture medium, and the suspension was centrifuged. For odontoblast differentiation, hDPSCs were transitioned to a differentiation medium composed of α-MEM supplemented with 10% FBS, 0.1 µM dexamethasone, 5 mM β-glycerophosphate, 50 µg/mL ascorbic acid, and 10 ng/mL TGF- β1.

#### 2.6.2. Cell Counting and Scaffold Seeding

To ensure precise experimental conditions, hDPSCs were quantified using a Neubauer hemocytometer. Following trypsinization and resuspension, 10 µL of the cell suspension was loaded into the chamber. Cells were counted in the four corner squares under a bright-field microscope, and the average count was used to determine the cell concentration (cells/mL = average count × 10^5^).

For cell–material interaction studies, hDPSCs (passages 3–5) were seeded at a density of 10,000 cells per well in 96-well plates. After seeding, cells were allowed to attach overnight under standard culture conditions (37 °C, 5% CO_2_). On the following day, PLGA and PLGA–fluorapatite (PLGA–FAP) microscaffolds were introduced into each well, in direct contact with the cells, at a concentration of 2% (*w*/*v*) in culture medium. This setup enabled evaluation of cell viability, attachment, metabolic activity, and overall cell–material interactions under direct contact conditions.

For immunofluorescence (IF) analysis, hDPSCs were seeded at a density of 80,000 cells per well in 24-well plates containing round glass coverslips at the bottom. After overnight attachment under standard culture conditions (37 °C, 5% CO_2_), cells were fixed using 4% paraformaldehyde (PFA) and subsequently processed for immunofluorescence staining and microscopic analysis.

### 2.7. Live Dead Staining and Epi-Fluorescent Microscopy

The viability of hDPSCs cultured on PLGA microscaffolds coated with different concentrations of FAP was qualitatively evaluated using a Live/Dead fluorescence staining assay. The PLGA microscaffolds were functionalized with FAP suspensions at concentrations of 0.5%, 0.25%, 0.1%, 0.05%, and 0.01% (*w*/*v*), corresponding to 5 mg/mL, 2.5 mg/mL, 1 mg/mL, 0.5 mg/mL, and 0.1 mg/mL, respectively. The resulting scaffolds were designated as mic_FAP_5, mic_FAP_2.5, mic_FAP_1, mic_FAP_0.5, and mic_FAP_0.1, according to their fluorapatite content. These labels do not represent quantified FAP content within the final microscaffold. A control group consisting of PLGA microscaffolds without FAP (mic_FAP_0) and a tissue culture polystyrene control (TCPS) were also included.

For the assay, hDPSCs were seeded onto the scaffolds at a final microscaffold concentration of 2% (*w*/*v*) in culture medium, in accordance with ISO 10993-5:2009 (International Standard confirmed, ISO/TC 194 https://www.iso.org/fr/standard/36406.html, accessed on 4 August 2026) [24], guidelines for the biological evaluation of the cytotoxicity of medical materials. The cells were cultured under standard conditions (37 °C, 5% CO_2_) and maintained in direct contact with the scaffolds. After 3 days of incubation, cell viability was assessed using a Live/Dead staining kit containing fluorescent viability indicators that distinguish viable and non-viable cells based on membrane integrity and esterase activity.

Following staining, the samples were incubated according to the manufacturer’s instructions and subsequently washed with sterile phosphate-buffered saline (PBS). The stained cells were then examined using a fluorescence microscope to visualize the distribution and viability of hDPSCs on the scaffold surface. Viable cells emitted green fluorescence, while dead cells appeared red, allowing qualitative evaluation of cytotoxicity and cell attachment on the different FAP-functionalized microscaffolds. Images were captured at 4×, 10×, and 40× magnifications to observe cell morphology and spatial distribution across the scaffold structures.

For quantitative analysis, three biological replicates per group were analyzed. Live and dead cells were quantified using ImageJ/FIJI Version 1.54p (https://imagej.net/) threshold-based cell counting. The percentage of viable cells was calculated and used for statistical analysis. Quantitative Live/Dead data were analyzed using GraphPad Prism (version 8.0.2, GraphPad Software, San Diego CA, USA). One-way ANOVA followed by Dunnett’s multiple comparison test was performed to compare all groups against the control group (mic_FAP_0 and/or TCPS, depending on comparison). Results were expressed as mean ± standard deviation.

### 2.8. Metabolic Activity by Alamar Blue Assay

To quantitatively evaluate the metabolic activity of DPSCs cultivated in direct contact with the formulated microscaffolds, an Alamar Blue (resazurin-based) cell viability assay was performed. The non-toxic, cell-permeable resazurin dye acts as an oxidation-reduction indicator; it is reduced by mitochondrial enzymes in metabolically active cells from a blue, non-fluorescent state to a pink, highly fluorescent compound (resorufin) [25]. The metabolic reduction was quantified by measuring the absorbance at 570 nm and 600 nm using a spectrometer at predetermined time points (Day 1, Day 3, and Day 7). At each time point, 10 µL of Alamar Blue reagent was added to 100 µL of culture medium per well, corresponding to a final concentration of 10% (*v*/*v*). The plates were incubated for 1 h at 37 °C in 5% CO_2_ protected from light before absorbance was measured at 570 nm and 600 nm. In accordance with ISO 10993-5 (2009) [24] guidelines, the microscaffold concentration was standardized to 2% (*w*/*v*) across all experimental groups (mic_FAP_0.1 to mic_FAP_5) and controls (mic_FAP_0 and TCPS). Data were analyzed using two-way ANOVA to evaluate the effects of time and material condition, followed by Dunnett’s multiple comparison test. TCPS and mic_FAP_0 were used as control groups. Results were expressed as mean ± standard deviation. Independent wells were used for each time point (Days 1, 3, and 7), with measurements performed in triplicate for each experimental condition.

### 2.9. Immunofluorescence Assay

Immunofluorescence staining was performed to evaluate the expression of specific cellular markers in hDPSCs cultured under different experimental conditions. Only the 1% and 2.5% FAP formulations were selected as representative intermediate-to-higher FAP conditions that retained favorable cytocompatibility and cell–material interaction in the screening assays, while avoiding the 5% condition that showed reduced viability and greater aggregation. The comparison was intended as a focused exploratory assessment of marker expression rather than a full concentration-response analysis.

Cells were seeded onto glass coverslips and allowed to attach overnight under standard culture conditions (37 °C, 5% CO_2_). Following incubation, samples were fixed using 4% PFA and mounted on glass slides. After fixation, samples were treated with PBS containing 0.2% Tween-20 for 15 min to facilitate permeabilization. Subsequently, non-specific binding sites were blocked by incubating the samples in a blocking solution consisting of 0.5% bovine serum albumin (BSA) and 0.1% Triton X-100 in PBS (PBS-BT) for 15 min at room temperature. Primary antibodies against SOX2, RUNX2, CD90, and Vimentin were diluted in blocking solution and incubated with the samples for 1 h at room temperature. The following primary antibodies were used: goat anti-human SOX2 antibody, diluted 1:250 (catalogue number: sc-17320, Santa Cruz Biotechnology, Heidelberg, Germany); mouse anti-human Vimentin antibody, diluted 1:250 (Dako); rabbit anti-human RUNX2 antibody, diluted 1:250 (catalogue number: ab192256, Abcam, LubioScience GmbH, Zurich, Switzerland; and mouse anti-human CD90 antibody, diluted 1:250 (catalogue number: ab181469, Abcam, LubioScience GmbH, Zurich, Switzerland). After primary antibody incubation, samples were washed three times with PBS and incubated with the appropriate species-specific fluorescent secondary antibodies diluted 1:250 in blocking solution for 1 h at room temperature protected from light. The following secondary antibodies were used: Alexa Fluor 568 donkey anti-goat IgG secondary antibody, diluted 1:250 (catalogue number: A11057, Invitrogen, Thermo Fisher Scientific, Reinach BL, Switzerland); Alexa Fluor 647 donkey anti-mouse IgG secondary antibody, diluted 1:250 (catalogue number: A31571, Invitrogen, Thermo Fisher Scientific, Reinach BL, Switzerland); Alexa Fluor 568 goat anti-rabbit IgG secondary antibody, diluted 1:250 (catalogue number: A11036, Invitrogen, Thermo Fisher Scientific, Reinach BL, Switzerland) and Alexa Fluor 647 donkey anti-mouse IgG secondary antibody, diluted 1:250 (catalogue number: A31571, Invitrogen, Thermo Fisher Scientific, Reinach BL, Switzerland).

For cytoskeletal visualization, samples were further incubated with phalloidin solution for 15 min at room temperature. Phalloidin stock solution was prepared by dissolving it in dimethyl sulfoxide (DMSO) and subsequently diluting it to 1:1000 in PBS containing 1% BSA. After incubation, samples were washed twice with PBS for 5 min each. Nuclear staining was performed using 4′,6-diamidino-2-phenylindole (DAPI), applied as one drop per sample. Finally, samples were mounted using a suitable mounting medium and covered with a glass coverslip. Fluorescent images were acquired using an epifluorescence microscope (Leica DM4000B, Wetzlar, Germany) under appropriate filter settings. All immunofluorescence experiments were performed using three independent biological replicates per experimental condition. Representative images were selected from these independent experiments, and fluorescence quantification was normalized to the DAPI signal before statistical comparison.

### 2.10. Statistical Analysis

Statistical analysis was performed using GraphPad Prism (version 8.0.2, GraphPad Software, San Diego CA, USA). All quantitative data are presented as the mean ± standard deviation (SD) from at least three independent experiments. For electrical conductivity measurements, one-way analysis of variance (ANOVA) followed by Dunnett’s multiple comparison test was used. The control group was defined as 0% (*w*/*v*) FAP for PLGA–FAP suspensions and double-distilled water (ddH_2_O) for FAP suspension measurements. Metabolic activity and cell viability data were analyzed using two-way ANOVA to assess the effects of time and treatment conditions, followed by Dunnett’s multiple comparison test. Control groups included TCPS and mic_FAP_0 where appropriate. A *p*-value < 0.05 was considered statistically significant.

## 3. Results

### 3.1. PLGA–Fluorapatite Microscaffolds Exhibit Concentration-Dependent Changes in Surface Morphology and Porosity

**Figure 2:**
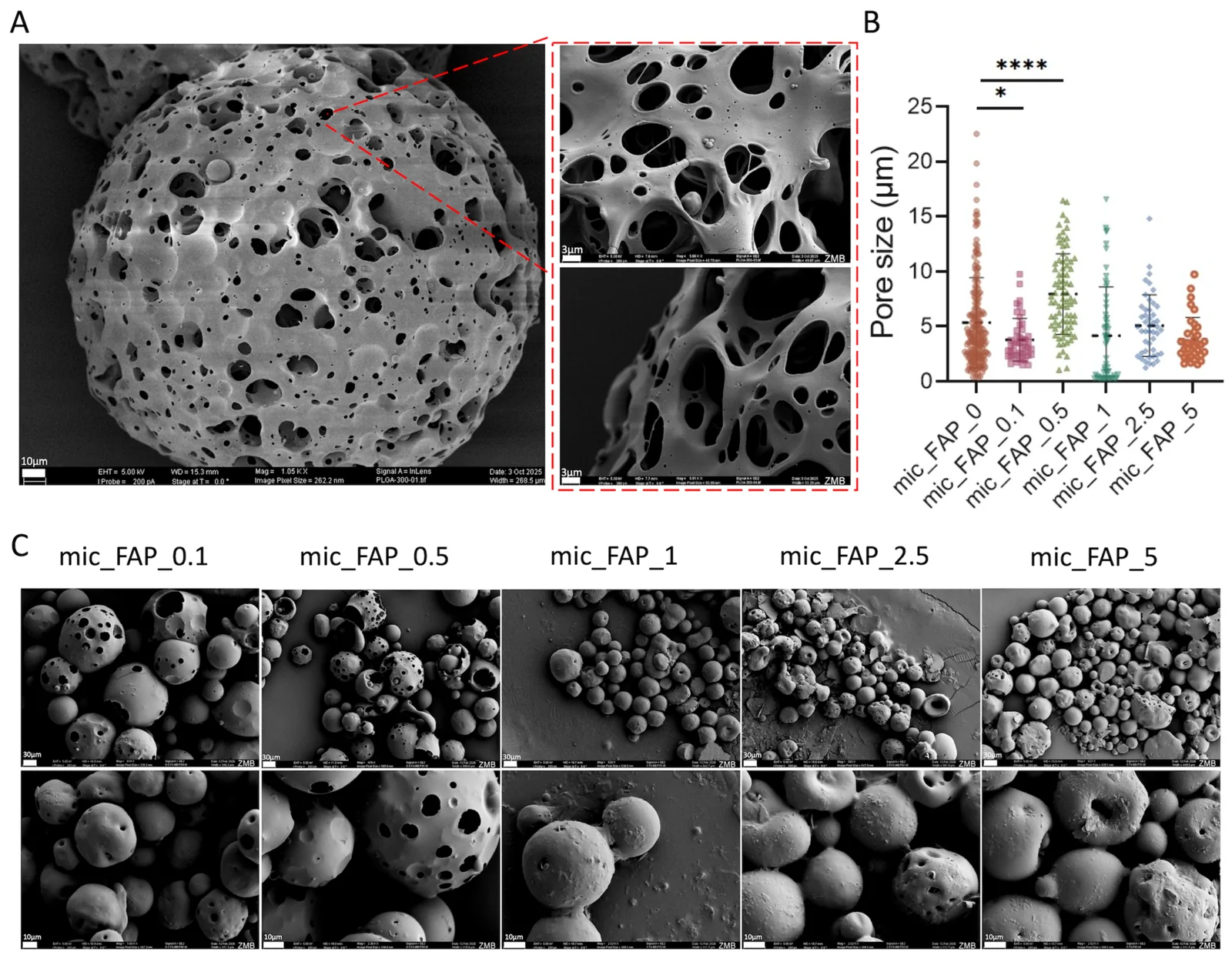
Morphological characterization of fluorapatite-loaded PLGA–PVA microscaffolds. (**A**) Scanning electron microscopy (SEM) images of PLGA–PVA microscaffolds, illustrating surface morphology and porous structure. (**B**) Quantitative analysis of microscaffold porosity, presented as a graph. (**C**) SEM images of PLGA–PVA microscaffolds loaded with varying concentrations of FAP, shown at two different magnifications, highlighting changes in surface morphology and porosity upon FAP incorporation. Statistical significance between the experimental groups with the control group is indicated as * *p* < 0.05 and **** *p* < 0.0001.

To evaluate the structural morphology, surface topography, and porous architecture of the synthesized PLGA microscaffolds before and after functionalization with varying concentrations of fluorapatite, SEM was performed (Figure 2). The pristine control microscaffolds (PLGA Control) displayed a highly uniform, spherical geometry with a well-controlled size distribution, where over 75% of the fabricated spheres possessed diameters ranging between 50 and 200 µm (Figure 2A). Quantitative pore-size analysis was performed, and values are presented as mean ± standard deviation (SD). The number of pores measured per group was for all experimental groups at least 50. One-way ANOVA followed by Dunnett’s multiple comparisons test was used to compare each FAP-loaded group with the mic_FAP_0 control group.

Measuring the individual surface pore diameters across many experimental groups revealed that FAP loading exerts a dynamic, non-linear influence on matrix density and structural arrangement (Figure 2B,C); the (mic_FAP_0) had a markedly heterogeneous pore distribution. The median pore size was approximately 5.3 μm, with numerous massive macro-pores reaching into the 20–25 μm range.

The (mic_FAP_0.1) triggered an initial compaction of the polymer matrix. The incorporation of trace FAP considerably limited the pore network, reducing the median diameter to roughly 3.8 μm (* *p* < 0.05) and greatly constricting the overall distribution compared to the control. (mic_FAP_0.5) markedly inverted this constricting trend. At this particular concentration, the median pore size increased to its maximum value among all groups, approximately 8.0 μm (**** *p* < 0.0001) (Figure 2A,B). This indicates that FAP loading did not produce a strictly linear pore-size response. Instead, the intermediate mic_FAP_0.5 concentration generated the most open surface pore architecture, while higher FAP concentrations appeared to reduce visible pore accessibility. The architectural transformation is clearly evident in the SEM micrographs (Figure 2C), which exhibit significant, elongated surface cavities and macro-voids.

In (mic_FAP_1 to mic_FAP_5), increasing the FAP concentration resulted in a gradual, concentration-dependent enhancement of structural refinement. Both the mic_FAP_1 and mic_FAP_2.5 formulations exhibited median pore diameters adjusted to a narrower range of 4.0–5.0 μm, while preserving a substantial density of micro-voids. Upon reaching maximum loading capacity (mic_FAP_5), the surface exhibited significant uniformity and constraint, resulting in a median pore size of around 3.5 μm, with no presence of huge outlier macro-pores (>10 μm) (Figure 2B,C).

### 3.2. FTIR Characterization Demonstrated Preservation of PLGA Structure with Successful FAP Surface Modification

**Figure 3:**
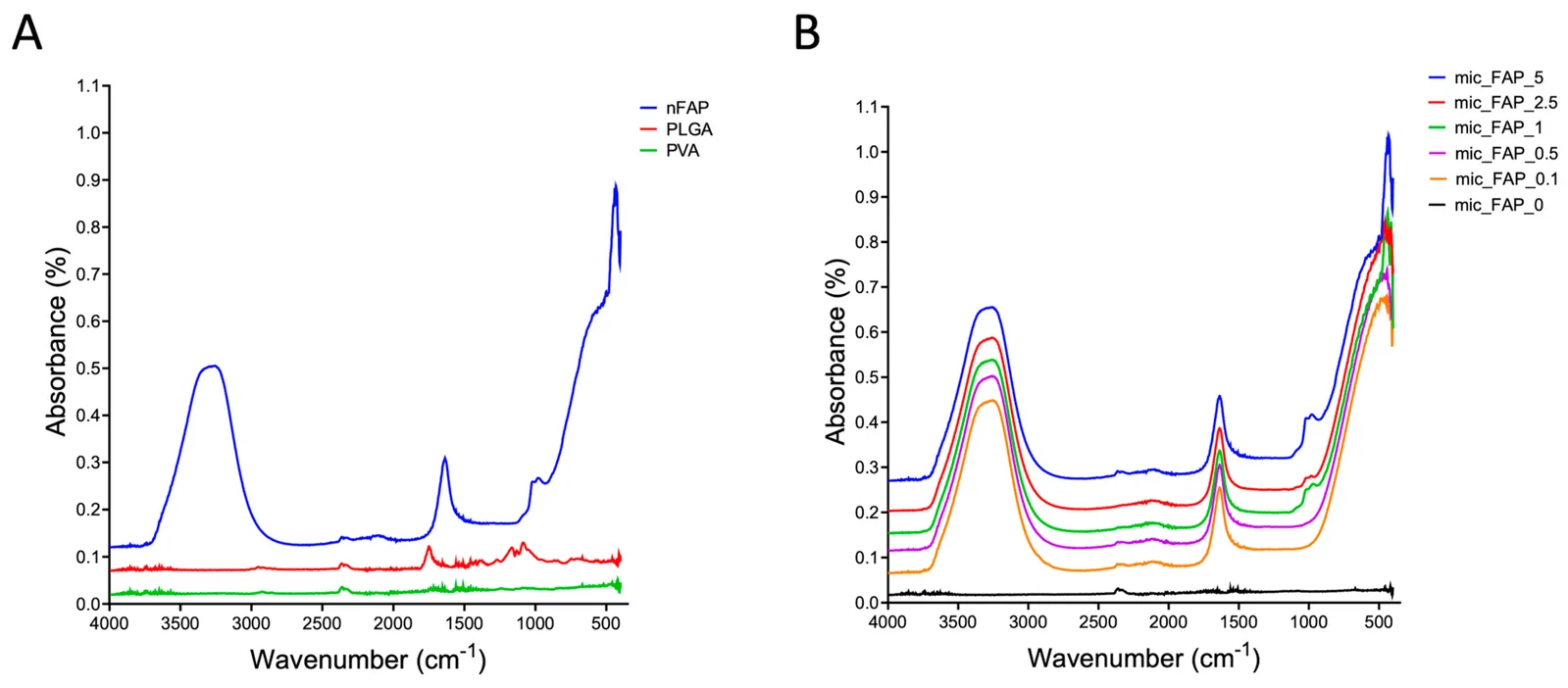
Fourier-transform infrared (FTIR) analysis of base materials and fabricated microscaffolds. (**A**) FTIR spectra of the base components, including poly(lactic-co-glycolic acid) (PLGA), polyvinyl alcohol (PVA), and FAP, showing their characteristic functional groups. (**B**) FTIR spectra of PLGA–PVA microscaffolds loaded with varying concentrations of fluorapatite, confirming successful incorporation of FAP into the polymeric matrix through the presence of characteristic peaks and potential shifts in absorption bands.

FTIR spectroscopy verified the chemical composition of the unmodified PLGA/PVA matrices and confirmed successful surface modification across all FAP concentration groups are shown in (Figure 3A). The baseline spectrum of the control microscaffolds (mic_FAP_0) exhibited the definitive fingerprint bands of both constituent polymers. The PLGA backbone was identified by the strong ester carbonyl (C=O) stretch at 1750–1745 cm^−1^, asymmetric/symmetric C–H stretching at 2995–2945 cm^−1^ and 2880–2850 cm^−1^, and regional C–O–C/C–O stretching at 1265–1045 cm^−1^. The co-existence of residual PVA surfactant was verified by the broad O–H stretching envelope at 3600–3200 cm^−1^ and a crystalline C–O stretching peak at 1145–1085 cm^−1^ (Figure 3A). In comparison, the spectrum of pure FAP powder was characterized by distinct phosphate functional group vibrations. These included the triply degenerate asymmetric PO_4_^3−^ stretching mode (ν_3_) at 1000–1100 cm^−1^ (with a distinct shoulder at ~1090–1100 cm^−1^), the symmetric PO_4_^3−^ stretch (ν_1_) at ~959 cm^−1^, and well-resolved dual peaks for the triply degenerate PO_4_^3−^ bending modes (ν_4_) at ~600 and 560 cm^−1^. Composite scaffolds functionalized with 0.1 to 5 mg/mL FAP suspensions, displayed clear spectral superimposition of the underlying polymer and the newly integrated mineral phase. Crucially, the signature PLGA ester carbonyl peak (~1750 cm^−1^) remained entirely unaltered in position and definition across all functionalized groups, demonstrating that the structural and chemical integrity of the host polymeric scaffold was fully preserved throughout the mineral coating process (Figure 3B).

Furthermore, the successful incorporation of FAP was confirmed by the emergence of the characteristic phosphate bands within the composite spectra. As the concentration of the FAP suspension increased from (mic_FAP_0.1) to (mic_FAP_5), a corresponding dose-dependent increase in the intensity of the ν_3_ asymmetric PO_4_ stretch (1000–1100 cm^−1^) and the ν_4_ PO_4_ bending peaks (~600 cm^−1^ and ~560 cm^−1^) was observed. In the lower concentration groups (mic_FAP_0.1 and mic_FAP_0.5), the phosphate peaks appeared as subtle shoulders on the broader polymer C–O stretching bands. However, in the higher concentration groups (mic_FAP_2.5 and mic_FAP_5), the PO_4_^3−^ signals became highly pronounced, dominating the lower wavenumber region (Figure 3B). These concentration-dependent spectral changes are consistent with increased surface-associated phosphate-containing mineral, but the actual mass fraction of adsorbed FAP was not quantified.

### 3.3. FAP-Loaded PLGA Microscaffolds Showed Enhanced Conductive Properties Compared with Unloaded Controls

**Figure 4:**
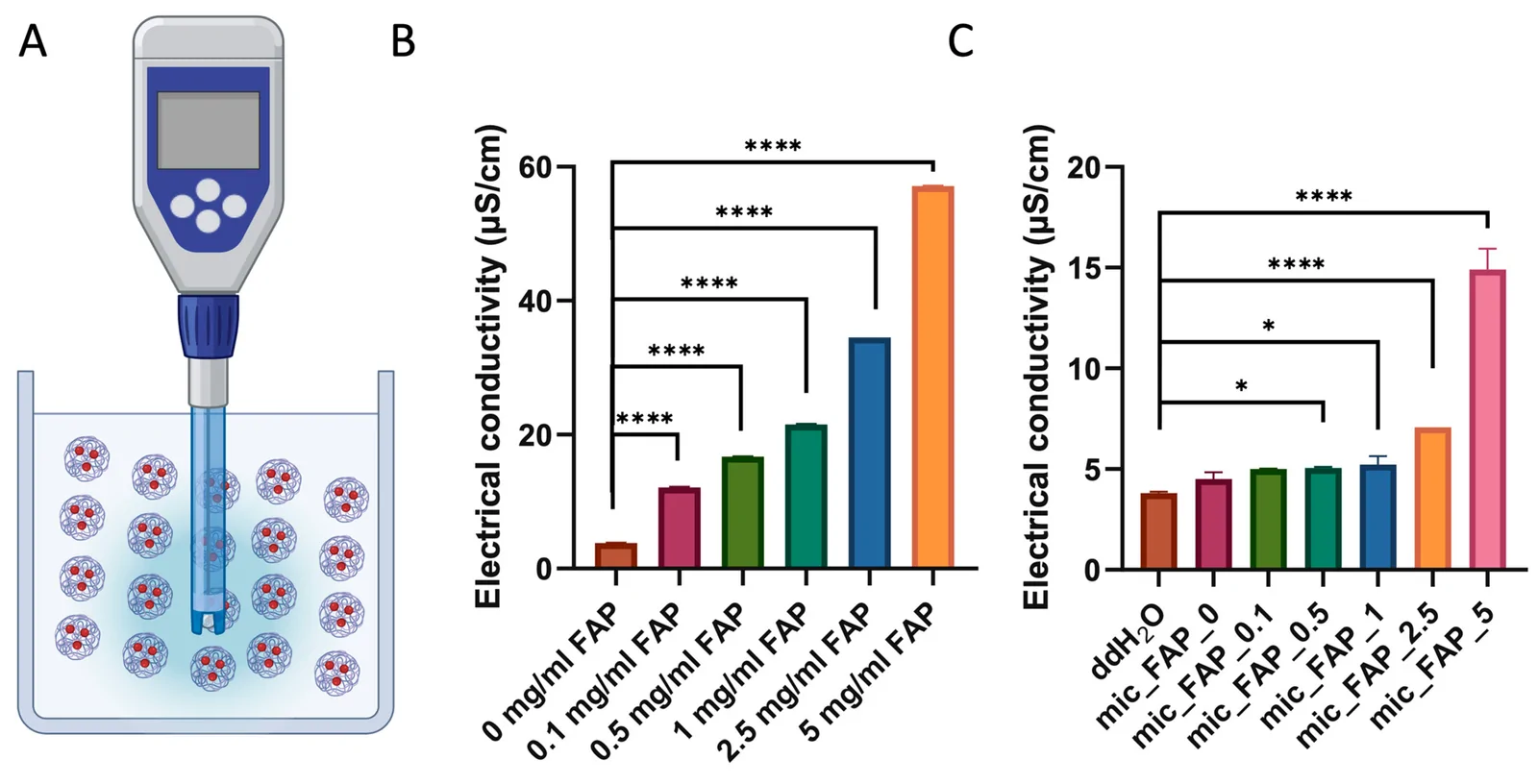
Electrical conductivity of fluorapatite suspensions and microscaffold formulations. Graphical representation of the apparatus and measurement process. (**A**) Electrical conductivity (µS/cm) of FAP suspensions alone. Suspensions of biofabricated PLGA–PVA microscaffolds loaded with varying concentrations of FAP in double-distilled water (ddH_2_O). (**B**,**C**) Collected and analyzed conductivity data are presented as mean ± standard deviation. Statistical significance between the experimental groups with the control group is indicated as * *p* < 0.05 and **** *p* < 0.0001.

To characterize the electrochemical properties and ionic behavior of both the precursor mineral components and the final composite formulations, electrical conductivity measurements were systematically executed (Figure 4A).

As shown in Figure 4B,C, electrical conductivity increased significantly with increasing FAP concentration in both FAP suspensions and FAP-loaded PLGA-PVA microscaffold suspensions. FAP suspensions alone exhibited conductivity values ranging from 3.80 μS/cm (0 mg/mL) to 57.13 μS/cm (5 mg/mL). Ordinary one-way ANOVA coupled with Dunnett’s multiple comparisons test confirmed that this mineral-driven ionic enhancement was highly significant across all evaluated thresholds (*p* < 0.0001), establishing a direct, proportional relationship between FAP particle loading and the concentration of freely suspended, electrochemically active ionic species within the aqueous phase. (Figure 4B).

Similarly, all biofabricated PLGA-PVA microscaffolds loaded with FAP concentrations showed significantly higher conductivity than the control group (0 mg/mL FAP; *p* < 0.0001). In microscaffold suspensions, conductivity increased from 4.51 μS/cm in unloaded microscaffolds to 14.91 μS/cm in microscaffolds containing 5 mg/mL FAP. While unloaded microscaffolds did not differ significantly from ddH_2_O (*p* = 0.2488), all FAP-loaded microscaffold groups showed significantly increased conductivity, with the strongest effects observed at 2.5 and 5 mg/mL FAP (*p* < 0.0001) (Figure 4C).

A concentration-dependent increase in aqueous electrical conductivity was observed with increasing FAP treatment concentration. These measurements are interpreted as a physicochemical property of the suspensions under the tested ddH_2_O conditions and not as direct evidence of physiological electrochemical activity or regenerative bioactivity.

### 3.4. Live/Dead Staining Demonstrated Favorable Cytocompatibility of PLGA–FAP Microscaffolds

**Figure 5:**
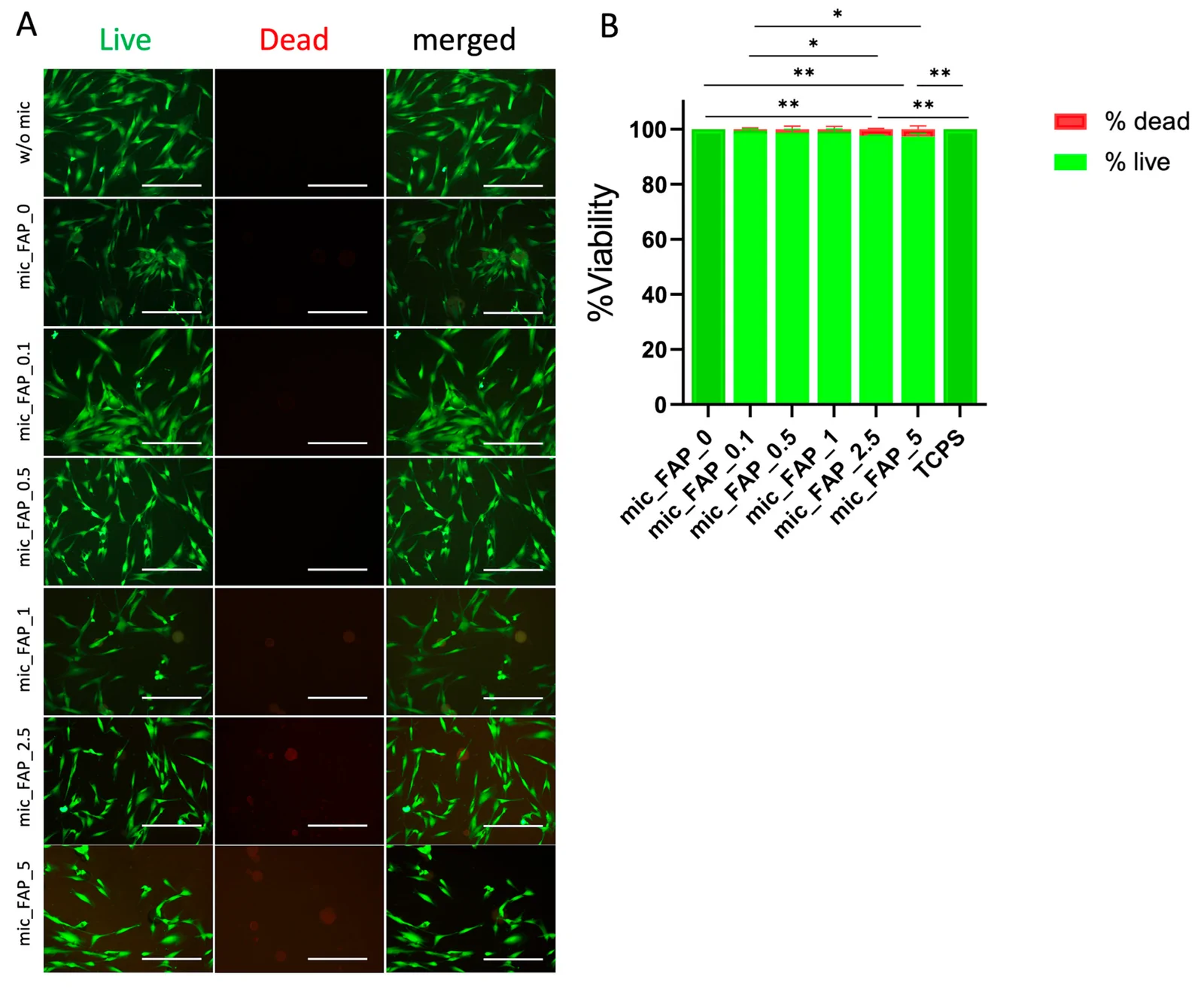
Cytotoxicity assessment of DPSCs cultured with fluorapatite-loaded microscaffolds after 1 day in culture. Cytotoxicity of DPSCs in direct contact with PLGA–PVA microscaffolds containing varying concentrations of fluorapatite was evaluated using Live/Dead staining. (**A**) Representative fluorescence images show viable (live, in green) and non-viable (dead, in red) cells after incubation. (**B**) Quantitative analysis of cell viability was performed using ImageJ software. Experiments were conducted in triplicate with three independent replicates per group. Data are presented as mean ± standard deviation. Statistical significance is indicated as * *p* < 0.05, ** *p* < 0.01. Scale bar represents 50 µm. The different shades of green reflect minor variations in cell viability, with darker green indicating viability values close to or above 99%.

**Figure 6:**
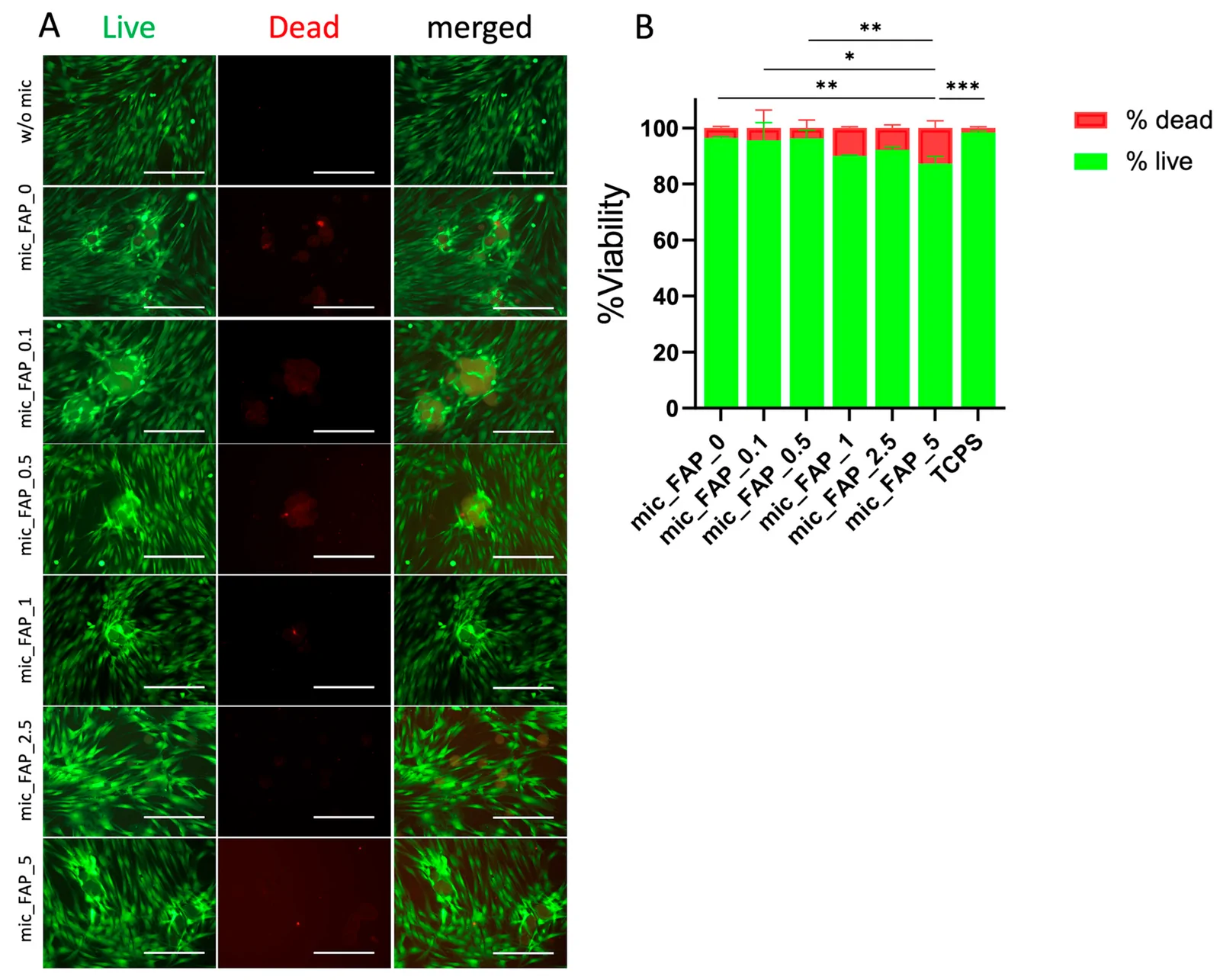
Cytotoxicity assessment of DPSCs cultured with fluorapatite-loaded microscaffolds after 3 days in culture. Cytotoxicity of DPSCs in direct contact with PLGA–PVA microscaffolds containing varying concentrations of fluorapatite was evaluated using Live/Dead staining. (**A**) Representative fluorescence images show viable (live) and non-viable (dead) cells after incubation. (**B**) Quantitative analysis of cell viability was performed using ImageJ software. Experiments were conducted in triplicate with three independent replicates per group. Data are presented as mean ± standard deviation. Statistical significance is indicated as * *p* < 0.05, ** *p* < 0.01, *** *p* < 0.001. Scale bar represents 50 µm. The different shades of green reflect minor variations in cell viability, with darker green indicating viability values close to or above 99%.

Live/Dead staining demonstrated high viability of DPSCs cultured with FAP-loaded PLGA–PVA microscaffolds at both Day 1 and Day 3. Fluorescence images revealed predominantly live (green) cells with only sparse dead (red) cells in all groups (Figure 5A and Figure 6A). On Day 1, no significant differences were observed between mic_FAP_0 and mic_FAP_0.1, mic_FAP_0.5, or mic_FAP_1 (*p* > 0.05), whereas mic_FAP_2.5 and mic_FAP_5 exhibited significantly reduced viability (*p* = 0.0038 and *p* = 0.0014, respectively) (Figure 5B).

Extended incubation for up to 3 days demonstrated an advancement in the cellular response, highlighting the continuous viability of the culture system (Figure 6A). Quantified fluorescence statistics at day 3 demonstrated that the mic_FAP_0.1, mic_FAP_0.5, mic_FAP_1, and mic_FAP_2.5 formulations exhibited strong, stable viability across time, with no statistically significant differences when compared to the pristine mic_FAP_0 control (*p* > 0.12). However, the group with the highest mineral saturation (mic_FAP_5) exhibited a more significant divergence during the 3-day period. Analysis of multiple comparisons indicated a significant reduction of 9.16% in the viable cell ratio for mic_FAP_5 compared to mic_FAP_0 (*p* = 0.0082), which corresponded to a proportionate 9.16% increase in the dead cell subpopulation (*p* = 0.0082) (Figure 6B).

**Figure 7:**
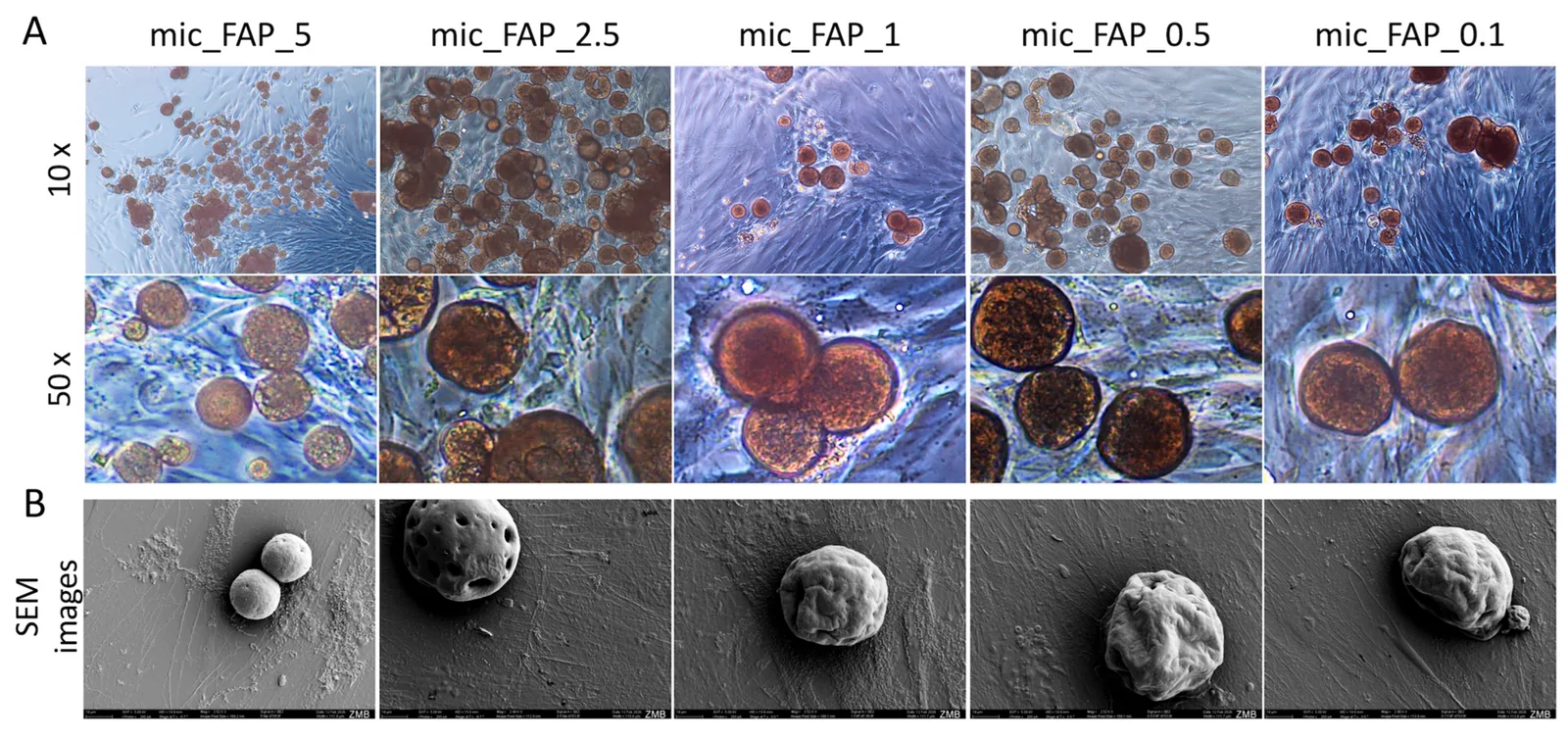
Evaluation of cell morphology in direct contact with microscaffolds. Optical microscopy images of hDPSCs cultured with different fabricated PLGA–PVA microscaffolds containing varying concentrations of fluorapatite after 3 days. (**A**) Cell morphology and attachment were assessed at two magnifications: low magnification (1×, upper panel) and high magnification (50×, lower panel). (**B**) SEM micrographs obtained at the same time point further demonstrate cell attachment, spreading, and direct interaction with the microscaffold surface morphology. Scale bar in SEM images: 10 µm. The images demonstrate cell spreading and interaction with the microscaffold surfaces.

To correlate these viability profiles with physical cell behavior, optical microscopy (Figure 7A) and high-resolution SEM (Figure 7B) were deployed at Day 3 to evaluate morphological adaptations in direct contact with the micro-matrices. As illustrated in (Figure 7A), the DPSCs exhibited extensive cellular spreading, active migration, and direct structural interaction with the microscaffold boundaries across all examined concentrations. At lower magnifications (10×), continuous cellular sheets were observed anchoring multiple adjacent spheres together. High-magnification optical imaging (50×) and complementary SEM microphotographs clearly resolved a classical fibroblastic, spindle-like morphology. Cells extended prominent cytoplasmic processes and filopodia that actively adhered to and flattened across the curved, mineralized topologies of the microscaffolds.

Additionally, Figure 7 demonstrates that at intermediate FAP loading-suspension concentrations (0.5–2.5 mg/mL; 0.05–0.25% *w*/*v*), cell distribution appeared more homogeneous with favorable cell-material interaction. Conversely, at the highest concentration (5 mg/mL; 0.5% *w*/*v*), greater microscaffold aggregation and reduced interparticle spacing were observed.

### 3.5. PLGA–FAP Microscaffolds Promoted Concentration-Dependent hDPSC Metabolic Activity and Growth

**Figure 8:**
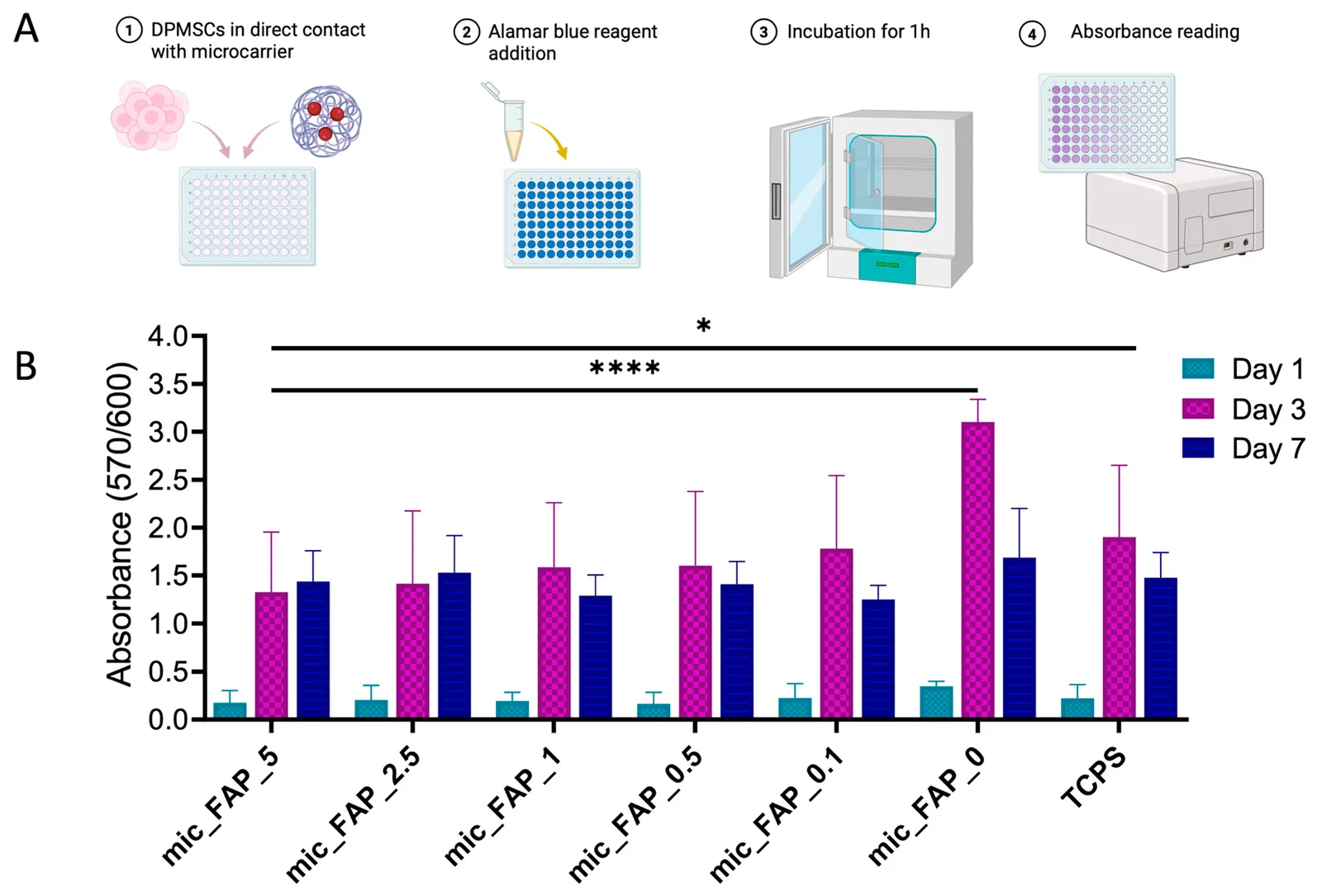
Metabolic activity of DPSCs in contact with fluorapatite-loaded microscaffolds. (**A**) Graphical representation of the experimental process. (**B**) Metabolic activity of DPSCs was evaluated using the Alamar Blue assay after direct contact with PLGA–PVA microscaffolds containing varying concentrations of fluorapatite at days 1, 3, and 7. Two control groups were included: microscaffolds without fluorapatite (PLGA–PVA only) and TCPS as a standard culture control. Data are presented as mean ± standard deviation. Statistical significance is indicated as * *p* < 0.05 and **** *p* < 0.0001.

An Alamar Blue experiment was conducted over a 7-day culture period to assess the metabolic progression of DPSCs on FAP-functionalized scaffolds (Figure 8A).

On day 1, all comparisons versus TCPS were non-significant with adjusted *p* values ranging from 0.9775 to >0.9999, and all comparisons versus mic_FAP_0 were also non-significant with adjusted *p* values ranging from 0.8851 to 0.9796 (Figure 8B). By Day 3, a significant, multi-faceted increase in metabolic activity was noted in all cultures, indicating active cell division. The highest response was observed in mic_FAP_0. When mic_FAP_0 was used as the control, all fluorapatite-containing formulations showed significantly lower absorbance, including mic_FAP_0.1, mic_FAP_0.5, mic_FAP_1, mic_FAP_2.5, and mic_FAP_5, and TCPS was also significantly lower than mic_FAP_0 (all adjusted *p* < 0.0001). When TCPS was used as the control, only the mic_FAP_5 group showed a statistically significant reduction in metabolic activity relative to TCPS (mean difference 0.5743, 95% CI 0.0387 to 1.110, adjusted *p* = 0.0303), whereas mic_FAP_0.1, mic_FAP_0.5, mic_FAP_1, and mic_FAP_2.5 did not differ significantly from TCPS. In contrast, mic_FAP_0 showed significantly greater absorbance than TCPS on day 3 (mean difference 1.200, 95% CI 0.6643 to 1.736, adjusted *p* < 0.0001) (Figure 8B). At day 7, the differences observed at day 3 were no longer present. No significant differences were found between TCPS and any microscaffold group, and no significant decrease in metabolic activity was found between mic_FAP_0 and any fluorapatite-loaded formulation or TCPS (Figure 8B).

### 3.6. PLGA–FAP Microscaffolds Enhanced Expression of Stemness and Differentiation-Associated Markers

**Figure 9:**
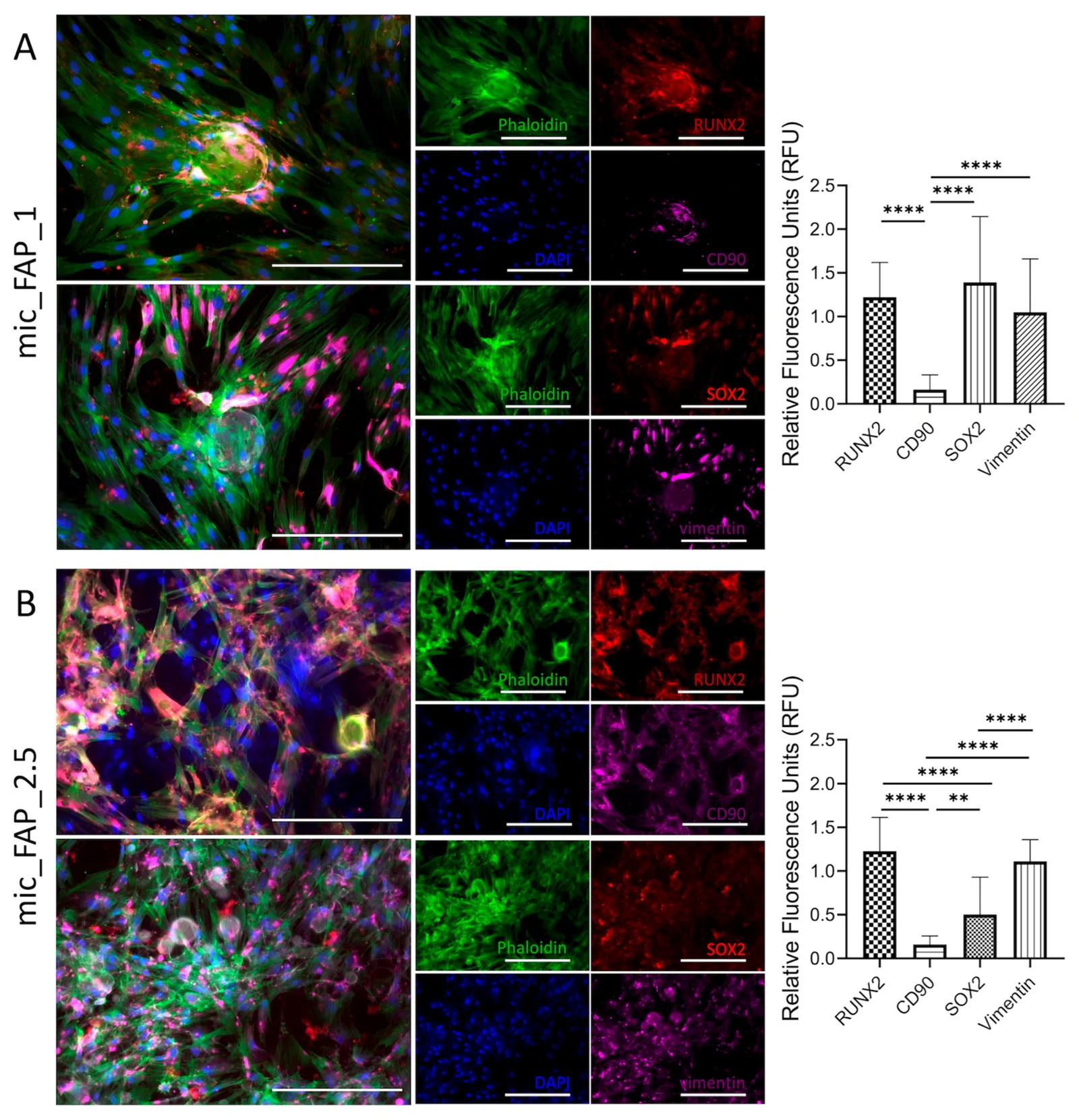
Immunofluorescence characterization of hDPSCs cultured on PLGA–FAP scaffolds at different concentrations. Immunofluorescence staining of hDPSCs cultured on PLGA scaffolds functionalized with (**A**) 1 mg/mL FAP and (**B**) 2.5 mg/mL FAP. Cytoskeletal organization was visualized using Phalloidin (green), while cell nuclei were stained with DAPI (blue). Expression of osteogenic and stemness-related markers was evaluated, including RUNX2 (red), SOX2 (red), CD90 (magenta), and Vimentin (magenta). Merged images demonstrate the spatial distribution and co-localization of cytoskeletal structure, nuclear staining, and marker expression under different scaffold conditions. Quantification panels shown on the right side of (**A**,**B**) report the relative fluorescence units (RFU) measured using ImageJ and normalized to the DAPI signal. Statistical analysis was performed using one-way ANOVA. Significant differences are indicated as ** *p* < 0.01 and **** *p* < 0.0001. Scale bar represents 50 µm.

Immunofluorescence analysis was performed to evaluate morphological characteristics and marker expression of hDPSCs exposed to PLGA-FAP microscaffolds functionalized using 1 and 2.5 mg/mL FAP suspensions (0.1 and 0.25% *w*/*v*, respectively) (Figure 9).

In both groups, Phalloidin staining (green) revealed well-organized cytoskeletal structures, indicating successful cell attachment and spreading on the scaffold surfaces. DAPI staining (blue) confirmed the presence of a high number of viable cells with clearly defined nuclei, suggesting good cell viability under all conditions (Figure 9).

At 1 mg/mL FAP (0.1% *w*/*v*), hDPSCs exhibited robust, well-organized cytoskeletal networks characterized by extensive Phalloidin-positive stress fibers stretching across the matrix. Quantitative fluorescence analysis revealed that SOX2 and RUNX2 were the most predominantly expressed markers, yielding high relative fluorescence units (RFU) of approximately 1.4 and 1.2, respectively. Conversely, CD90 expression was remarkably low RFU, demonstrating a highly significant downregulation compared to both RUNX2 and SOX2. Vimentin displayed moderate-to-high intensity (~1.0 RFU), highlighting strong intermediate filament networks typical of these adherent populations, though its levels did not significantly differ from RUNX2 or SOX2 (Figure 9A).

At 2.5 mg/mL FAP (0.25% *w*/*v*) (Figure 9B), a more pronounced cell spreading and denser cellular network were observed compared to the 1% FAP group. While the overall architectural integration visualized via Phalloidin remained dense, the protein expression dynamics altered distinctly; RUNX2 maintained a consistently high level of expression (~1.2 RFU), establishing it as a highly stable marker across both experimental conditions. A pronounced drop was observed in SOX2 expression, which fell significantly to an RFU of approximately 0.5. This reduction was statistically significant when compared directly to RUNX2. CD90 remained consistently suppressed at negligible levels (~0.15 RFU), showing a stark, statistically significant difference against all other tested markers. Interestingly, Vimentin expression trended slightly higher in mic_FAP_2.5 (~1.1 RFU), becoming significantly more pronounced than both CD90 and SOX2 (Figure 9B).

## 4. Discussion

The present study investigated the effects of FAP functionalization on the properties of PLGA microscaffolds and evaluated in vitro the biological efficiency of the platform in hDPSC cultures. Our results demonstrated that, although mineral phase incorporation in the PLGA microscaffolds improved while increasing FAP loading, their biological performance was not directly proportional to the concentration of the incorporated FAP. Importantly, optimization rather than maximization of the fluorapatite loading based is essential for the maintenance of the microscaffold architecture and the induction of cellular responses.

PLGA has been extensively used as a synthetic polymer in drug delivery systems and tissue engineering due to its favorable characteristics regarding biomedical applications [26,27]. PLGA scaffolds with highly open, porous microarchitecture are structurally advantageous for tissue engineering applications, as they provide the essential spatial pathways required for unhindered nutrient diffusion, metabolic waste clearance, and initial cellular anchorage [13,14]. However, PLGA has limited intrinsic odontogenic properties and its inert surface limits stem cell adhesion and mineral deposition. This motivated us to functionalize the surface of PLGA microparticles using fluorapatite to improve cell-material interactions and provide biochemical signals that more closely resemble the mineralized extracellular matrix of dentin and bone [18,19]. Additionally, human dental pulp stem cells represent a highly significant cellular source for dentin and pulp regeneration [6,28,29]. Previous studies have shown that the success of pulp-dentin tissue engineering requires coordinated interaction between stem and progenitor cells, signaling factors, and a supportive scaffold microenvironment [6,30,31] that provides physical support and promotes cell adhesion, survival, migration, and differentiation. Earlier dental scaffold studies include PLGA/calcium-phosphate composites and FAP-modified PCL nanofibers, which evaluated hDPSC adhesion, proliferation, differentiation, and mineralization using three-dimensional scaffold systems and multiple differentiation/mineralization endpoints [31,32]. In contrast, the present study applies a post-fabrication, immersion-based FAP functionalization strategy to porous PLGA microscaffolds and compares a range of treatment concentrations across complementary physicochemical and short-term hDPSC response assays. This distinction allows assessment of how FAP treatment concentration influences the balance between visible surface pore morphology and cell compatibility in a microscale PLGA platform. It is also important to point out that the group nomenclature used throughout this study refers to the FAP concentration of the functionalization suspension (mg/mL) and not the actual amount of FAP adsorbed on the microscaffolds, which was not quantitatively determined. The present results therefore support a formulation-specific optimization of the FAP surface treatment, rather than a direct correlation between biological or physicochemical responses and the actual FAP content of the scaffolds.

Within the tested formulations, 2.5% FAP was considered the most promising concentration based on a multi-parameter comparison rather than on a single endpoint. It preserved accessible surface porosity, produced clear phosphate signals by FTIR, increased conductivity relative to the unloaded control, maintained favorable Live/Dead viability through Day 3, showed metabolic activity comparable to TCPS at Days 1 and 7, supported homogeneous cell spreading, and maintained RUNX2 expression in the immunofluorescence analysis. In contrast, 5% FAP produced the highest conductivity but was associated with greater aggregation, reduced pore accessibility, and a significant reduction in viability at Day 3. Therefore, optimal in this study refers to the best overall balance among the physicochemical and biological parameters evaluated, not proof of a universally optimal FAP concentration.

Similar formulation-dependent effects have been reported for PLGA/nHA scaffolds, where increasing nHA content reduced pore size, indicating that higher ceramic loading can limit surface pore accessibility [33]. However, other apatite-modified PLGA systems have shown increased pore size after ceramic modification, as reported for Sr/Zn-doped nHAp–PLGA composite scaffolds [34]. These findings support the interpretation that ceramic nanoparticle loading can alter visible pore morphology through competing mechanisms, including surface mineral deposition, polymer-particle interactions, and nanoparticle aggregation. Thus, the regenerative potential of composite scaffolds depends on achieving an appropriate balance between bioactivity and surface architecture, as excessive mineral loading may compromise the physical characteristics required for cell proliferation, viability and differentiation.

The non-linear pore-size response observed in the present study deserves particular emphasis. Relative to the ~5.3 µm median surface pore diameter of mic_FAP_0, mic_FAP_0.1 decreased to ~3.8 µm, whereas mic_FAP_0.5 transiently increased to ~8.0 µm before the pore diameter narrowed again at higher loading, reaching ~3.5 µm at mic_FAP_5. This pattern suggests that FAP deposition does not simply occlude pores in direct proportion to concentration. At low and intermediate loading, heterogeneous nanoparticle adsorption, local rearrangement of the hydrated polymer surface, and differences in particle distribution may transiently expose or enlarge visible surface openings, whereas at higher loading, aggregation and greater surface coverage are more likely to reduce pore accessibility. Because these measurements were obtained from SEM-visible surface pores rather than three-dimensional bulk porosity, the trend is interpreted as a concentration-dependent change in accessible surface architecture rather than a linear change in total scaffold porosity.

FTIR analysis confirmed the successful incorporation of fluorapatite into the PLGA/PVA scaffold matrix. The persistence of the characteristic PLGA carbonyl peak indicated that the polymer backbone remained chemically intact after functionalization, while the appearance and progressive increase in phosphate-related bands confirmed the presence of the apatite phase. This is important because an ideal surface modification strategy should enhance bioactivity without degrading the primary polymer structure. Fluorapatite is particularly attractive for oro-dental applications, as the substitution of hydroxyl groups with fluoride ions increases the crystallinity of apatite, reduces solubility, and improves resistance to acid dissolution compared to hydroxyapatite [18,19]. These characteristics may be particularly relevant in the oral environment, where biomaterials are exposed to pH fluctuations, bacterial byproducts, and inflammatory conditions. Furthermore, it has been previously reported that fluorapatite-modified surfaces promote the adhesion, differentiation, and mineralization of stem cells, including in dental pulp stem cell models [12,34,35].

The conductivity results further supported the concentration-dependent incorporation of fluorapatite into the scaffold system. Increasing FAP concentrations led to higher electrical conductivity in both the FAP suspensions and the FAP-loaded PLGA-PVA microscaffolds, suggesting a greater ionic contribution from the mineral phase. Although electrical conductivity is not typically considered the primary parameter in dentin and pulp regeneration, ion exchange and mineral availability are relevant to cell signaling, mineral nucleation, and hard tissue formation.

Consequently, the present findings showed that the highest conductivity does not represent the optimal biological performance. In this study, 5 mg/mL FAP condition exhibited the highest conductivity, but it was associated with the lowest visible surface pores, particle aggregation, and reduced cytocompatibility. These observations are important because they highlight the need to define an optimal bioactive range for the FAP nano-coating when seeking to enhance the regenerative potential of PLGA microscaffolds. Regarding the biological evaluation, a reduction in the metabolic activity observed in the 5% FAP group, was consistent with SEM observations, which showed aggregation and pore reduction, which could limit the cell spreading, nutrient diffusion, and available adhesion or interaction surfaces in the microscaffolds. Importantly, the spectra support the presence of FAP on the microscaffold surface after immersion-based adsorption, but they do not by themselves demonstrate formation of new covalent bonds or a specific chemical interaction between FAP and PLGA/PVA. The unchanged PLGA carbonyl band, together with the superimposed phosphate signals, is more consistent with physical surface incorporation/adsorption of the mineral phase while preserving the polymer backbone. Dedicated surface-chemical analyses would be required to establish specific interfacial bonding mechanisms. Accordingly, conductivity was used here as a physicochemical indicator of the ionic contribution associated with FAP functionalization, not as a stand-alone predictor of regenerative efficacy. The divergence between conductivity and biological performance at the highest FAP concentration indicates that increased ionic/electrical response must be balanced against pore accessibility, aggregation, and cytocompatibility.

These findings are consistent with previous reports indicating that mineralized or apatite-containing polymeric scaffolds can enhance the adhesion, proliferation, and differentiation of dental pulp stem cells, but that scaffold architecture and concentration-dependent effects of the material remain decisive determinants of the biological outcome [35,36]. In addition, the effect triggered by FAP coating on scaffolds morphology was not linear, as the transient increase in visible surface pore size at different concentrations can reflect a discontinuous nanoparticles coating which affect pore accessibility. Therefore, higher FAP treatment concentrations lead to nanoparticle aggregation and reduced accessibility of visible surface pores, resulting in a denser surface morphology which may also slightly reduce cell viability. In a recent study comparting HA and FAP-scaffolds, concluded that the biological advantages of FAP are dependent on the scaffold fabrication strategy and resulting in distinctive physicochemical properties, which translate into each FAP-containing biomaterial must be individually optimized and evaluated for its intended regenerative application [18].

Immunofluorescence analysis further supported the biological significance of the optimized concentration of FAP. hDPSCs cultured with 1% and 2.5% FAP maintained a mesenchymal phenotype, as suggested by the expression of vimentin and CD90, while testing positive for RUNX2, indicating early osteogenic/odontogenic commitment. RUNX2 is a key transcription factor involved in the differentiation of mineralized tissue and is commonly used as an early marker of osteogenic and odontogenic activation. The more pronounced cytoskeletal organization and cell extension observed with 2.5% FAP suggest improved cell-material interaction compared to 1% FAP. Interestingly, the maintenance of stemness-related markers alongside signals associated with early differentiation could indicate that 2.5% FAP creates a permissive microenvironment in which hDPSCs remain viable and plastic while beginning to activate mineralization-related pathways. This balance is particularly relevant for dentin and pulp regeneration, where premature or excessive mineralization may be undesirable, but controlled odontogenic activation is essential for the formation of reparative dentin. However, RUNX2 expression alone is not sufficient to demonstrate established odontogenic differentiation and further studies incorporating additional odontogenic markers, mineralization assays and longer differentiation periods will be required to confirm this response. These effects should then be validated in more sophisticated 3D and in vivo models to evaluate the regenerative potential of FAP-functionalized PLGA microscaffolds.

A limitation of the present study is that biological validation was primarily conducted under two-dimensional direct-contact culture conditions. In addition, the actual mass fraction and spatial distribution of FAP adsorbed onto the microscaffolds were not quantified, and conductivity was measured in ddH_2_O rather than a physiological electrolyte. FTIR provided supportive phosphate-related spectral evidence but did not independently establish fluorapatite phase identity or crystallinity. The immunofluorescence panel also represents early phenotype-associated markers and does not by itself demonstrate odontogenic differentiation or mineralized tissue formation. Future studies should therefore incorporate quantitative mineral-loading and release analyses, complementary mineral-phase characterization, three-dimensional pore characterization, additional odontogenic/mineralization endpoints, and advanced 3D, organotypic, and in vivo dentin-pulp models.

A recent study by Karatas et al. developed a multifunctional porous chitosan scaffold modified with Ti2NTx MXene and carbon quantum dots and demonstrated that scaffold performance must be assessed across complementary physicochemical and biological endpoints rather than inferred from a single material property [21]. Although that system targets wound healing and differs substantially from the present dental PLGA–FAP platform, it reinforces the broader design principle that biofunctional scaffold composition requires application-specific optimization of structure, material functionality, and cytocompatibility.

Nevertheless, our findings have important implications for the development of regenerative endodontic therapies. Current treatment of irreversible pulp damage relies predominantly on root canal therapy, which effectively eliminates infection but compromises the vitality of the tooth. Tissue engineering strategies seek to overcome this limitation by inducing natural mechanisms of repair through biomaterials that support the survival and function of resident stem cells, thereby enabling the regeneration of a functional dentin-pulp complex. Our study provides evidence that optimized PLGA-FAP microscaffolds can provide the structural support and bioactive microenvironment necessary to induce early cellular responses that promote tissue regeneration. Although further validation in advanced in vitro and in vivo models is required, these findings represent an important step toward the development of clinically translatable biomaterials for regenerative endodontics.

## 5. Conclusions

This study demonstrates that FAP surface functionalization induces concentration-dependent changes in the physicochemical properties and biological performance of porous PLGA microscaffolds cultured with hDPSCs, highlighting the importance of optimizing mineral incorporation. Among the tested conditions, 2.5 mg/mL FAP (0.25% *w*/*v*) provided the most favorable overall balance between surface pore morphology, phosphate-related FTIR signals, aqueous conductivity, cytocompatibility, and early cellular responses. These findings suggest that optimized FAP functionalization can enhance the properties of PLGA microscaffolds relevant to cellular support while preserving a porous architecture suitable for tissue-engineering applications. Collectively, FAP-functionalized PLGA microscaffolds represent a promising bioactive platform for further investigation in regenerative endodontics and dentin-pulp tissue engineering. Further studies, including quantitative FAP loading and mineral-phase characterization, odontogenic and mineralization assays, and validation in advanced 3D in vitro and in vivo models, are required to determine their potential to support functional dentin-pulp regeneration.

## Data Availability

The original contributions presented in this study are included in the article. Further inquiries can be directed to the corresponding author.
